# Performance of nitric acid and mineral enriched system versus phosphoric acid and universal adhesive in cervical carious lesions: a randomized clinical trial

**DOI:** 10.1038/s41405-025-00363-3

**Published:** 2025-09-01

**Authors:** Nourane Yasser Yassin, Dina Ezzeldin Mohamed, Olfat El Sayed Hassanein

**Affiliations:** 1 PhD candidate, Conservative Dentistry Department, Faculty of Dentistry, Cairo University, Egypt and Assistant lecturer, Conservative Dentistry Department, Faculty of Dentistry, Egyptian Russian University, Cairo, Egypt; 2 Lecturer of Conservative Dentistry, Conservative Dentistry Department, Faculty of Dentistry, Cairo University, Cairo, Egypt; 3 Professor of Conservative Dentistry, Conservative Dentistry Department, Faculty of Dentistry, Cairo University, Cairo, Egypt

**Keywords:** Bonded restorations, Aesthetic dentistry, Composite resin

## Abstract

**Objective:**

To evaluate the clinical performance of nitric acid associated with a mineral-enriched adhesive system compared to the conventional approach of phosphoric acid and a universal adhesive in cervical carious lesion restorations.

**Materials and methods:**

Twenty-six individuals with 44 cervical anterior carious lesions were randomized into two equal parallel groups. The intervention group received a nitric acid etch **(Clean and Boost dentin enamel cleanser, Vista Apex, USA)** in conjunction with a mineral-enriched adhesive and a flowable composite liner **(RE-GEN, Vista Apex, USA)**. Control group treated with phosphoric acid **(Scotchbond Universal Etchant, 3 M ESPE)**, a universal adhesive **(Single Bond Universal Adhesive, 3 M ESPE)**, and a standard liner **(Filtek Supreme, 3 M ESPE)**. All cavities were restored using nanohybrid resin composite **(Filtek Z350XT, 3 M ESPE)**. Postoperative sensitivity was assessed both qualitatively, using the modified USPHS criteria, and quantitatively, using a visual analog scale (VAS), as the primary outcome. In addition, clinical performance regarding marginal discoloration, adaptation, secondary caries, and retention was evaluated as secondary outcomes using modified USPHS criteria. Outcomes were assessed at 24 h, 6, 12, and 18 months. The data was statistically analyzed using intention-to-treat analysis. Intergroup comparisons were performed using the Chi-Squared test with a significance level (*p *≤ 0.05), and intragroup comparisons were analyzed using Cochran’s Q test with a confidence level of 95% and a study power of 80%. Relative risk was used to assess clinical significance. The survival rate was analyzed using the Kaplan-Meier and Log-rank tests. The study was conducted following the CONSORT 2025 guidelines.

**Results:**

Both groups demonstrated similar performance in terms of postoperative sensitivity, retention, secondary caries, and marginal adaptation, as assessed using modified USPHS criteria. However, there was a significant increase in marginal discoloration within the phosphoric acid groups after 18 months. There was 50% less risk of sensitivity with nitric acid compared to phosphoric acid using the VAS scale (CI (0.2512 to 0.9953); *p* = 0.0485). The tested groups showed an equal survival rate (*p* = 0.3771).

**Conclusion:**

Combining nitric acid with a mineral-enriched system is a promising approach for restoring cervical carious lesions.

## Introduction

Cervical caries presents one of dentistry’s most challenging and complex restorative problems, affecting the facial and/or lingual surface of posterior and anterior teeth [1]. The etiology of this lesion is complex and multifactorial; therefore, the dentist must identify and address the underlying cause before initiating the restorative procedure. These factors include the long-term accumulation of dental plaque on the teeth, a lack of proper oral hygiene, various gingival diseases, hormonal disturbances during pregnancy, exposure to certain medications that affect salivary secretion, and finally, the demineralization of enamel due to high consumption of carbohydrates and sugars [2]. Also, cervical carious lesions affect both enamel and dentin, complicating restoration due to the absence of materials that bond effectively to both substrates. The restoration of cervical carious lesions presents a considerable challenge for restorative dentists, due to complex clinical conditions that include difficult isolation, variations in tooth substrates, and intraoral biomechanical issues [3]. The risk of restoration failure increases by approximately 39% when both enamel and dentin are affected, in contrast to lesions confined to enamel. Moreover, several factors contribute to the success of restoring this condition, including the amount of sclerotic dentin, which increases with age, contamination of salivary and gingival fluids, and dentin hypersensitivity [1].

Another obstacle is selecting the best restorative material that can serve and survive in such challenging circumstances. Glass ionomer and its modifications were the material of choice due to their physicochemical properties, including bioactivity; however, they are not esthetically appealing nor mechanically long-lasting [4]. Hence, resin composites are widely utilized in restoring cervical lesions due to their bonding potential with enamel and dentin, which facilitates the preservation of intact tooth structure, optimal esthetics, and enhanced mechanical properties [5–7]. Nevertheless, failure may occur due to several challenges, such as the technique sensitivity of its placement, polymerization shrinkage, the type and design of the cavity, tooth location, the adhesive materials used, and operator- and patient-related factors. Although certain factors can be managed, others persist as inherent challenges [8].

Numerous modifications to resin composite materials and application techniques have been implemented and continue to be developed to address the aforementioned challenges [9]. The smear layer is an unstable structure that affects the chemical and mechanical bonding between the restoration and the tooth structure [10]. Different techniques to manage the smear layer include its complete removal, modification, or dissolution. The traditional technique for preparing the tooth substrate for resin composite restoration involves the complete removal of this layer using 35-37% phosphoric acid. However, postoperative sensitivity was a recurring issue reported by patients. A selective enamel etching technique was developed to address this issue by applying phosphoric acid exclusively to the enamel margins, followed by the application of a universal adhesive, also known as a multimode adhesive, to the entire prepared tooth structure [11]. This type of adhesive includes a mild acidic monomer to fulfill the purpose of dentin conditioning. However, subsequent research has demonstrated that the clinical performance of multimode adhesives is superior when used in the etch-and-rinse mode compared to the self-etch mode [12, 13]. Additionally, a commercial product has been developed to address the tooth substrate through an alternative method: the multifunction nitric acid etch. It contains acid, which facilitates etching and cauterizes minor bleeding; isopropyl alcohol serves as an antibacterial agent and surface cleaner prior to bonding; and Hydroxyethyl methacrylate (HEMA) is a desensitizer that blocks the dentinal tubules to reduce sensitivity [14].

Another factor that may enhance the performance of restorative systems is the incorporation of bioactive properties. The concept of bioactivity in dentistry involves using materials that interact with surrounding tissues to produce a beneficial biological response or release active substances that encourage remineralization, improve tissue health and regeneration, and extend the longevity of the restoration. Bioactivity was first introduced in the field of dentistry by Larry Hench in 1960. Bioactive materials exhibit various properties, including the inhibition of bacterial growth by releasing calcium, sodium, silica, and phosphate ions, as well as osteogenic properties attributed to calcium phosphate or tricalcium phosphate [15]. Limited trials have examined the impact of bioactive materials in restorative dentistry on biological, esthetic, and mechanical performance [16, 17].

The literature review revealed a lack of clinical trials examining the efficacy of multifunctional nitric acid etch in conjunction with mineral-enriched adhesive and flowable resin composite. Therefore, this study assessed the performance of nitric acid etch with a mineral-enriched adhesive and flowable resin composite compared with phosphoric acid etch using a universal adhesive and a regular flowable composite. The null hypothesis stated that there is no significant difference between the clinical performance of the two systems in terms of postoperative sensitivity (primary outcome), and marginal discoloration, marginal adaptation, secondary caries, and retention (secondary outcomes).

## Methods

### Study setting and design

The study was conducted at the Conservative Dentistry Department Outpatient Clinic, Faculty of Dentistry, Cairo University, from July 2023 to March 2025. The principal researcher (N.Y, experienced dentist) carried out all activities associated with the research project, including explaining and performing the procedures to the participants. The trial was designed as a parallel, two-armed, triple-blind study involving participants, assessors, and data analysts, with a 1:1 allocation ratio (regarding the number of lesions included in each arm). The trial is registered in the clinical trial registry site (www.clinicaltrials.gov) under the identification number (NCT05928533 on 26/06/2023). All required application forms, checklists, and informed consent documents were submitted to and approved by the Research Ethics Committee (REC) of the Faculty of Dentistry, Cairo University. These documents were subsequently submitted to the REC for approval to mitigate any ethical issues or potential harm to participants during the study. The study received approval and was assigned an ID number (11/7/23). The study was reported in accordance with the CONSORT 2025 guidelines [18].

### Sample size calculation

**Fig. 1:**
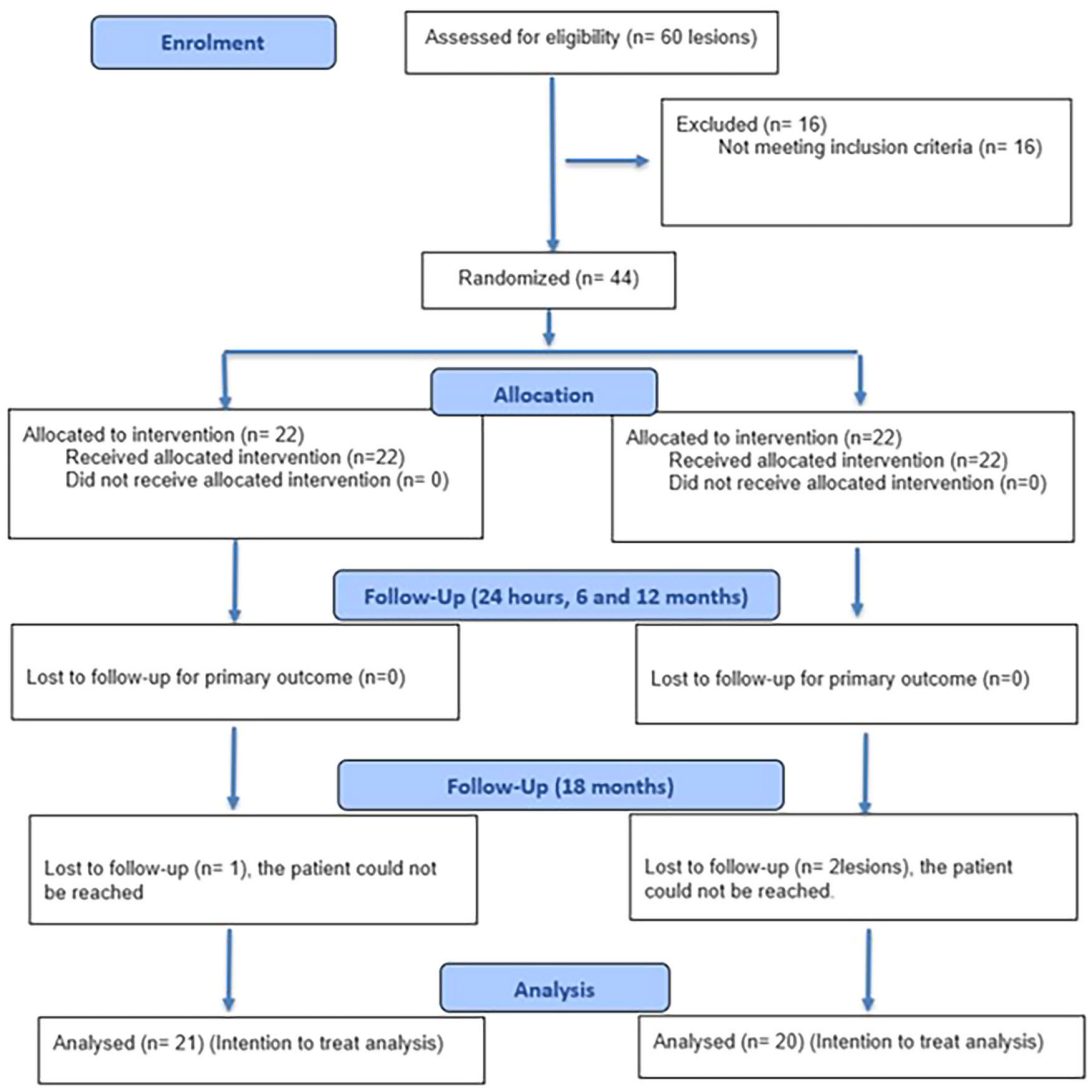
Flow diagram (Consort, 2025). Chart showing allocation of the enrolled participants.

A power analysis was conducted to ensure adequate power for statistical tests of the research hypothesis, comparing nitric acid and mineral-enriched adhesive with phosphoric acid and universal adhesive regarding postoperative sensitivity for cervical restorations after 18 months Fig. 1. The findings of Corral et al. [19] indicate that the phosphoric acid group exhibited a general distribution with a standard deviation of 1.11. The observed difference between the experimental and control means was 1, with a Cohen’s d effect size of 0.9. To reject the null hypothesis that the population means of the experimental and control groups are equal with a power of 0.8, a sample size of 17 teeth per group was required. The Type I error probability associated with this test of the null hypothesis was 0.05. The sample size was increased by 30% to account for potential dropouts, resulting in 22 per group. The sample size was calculated using PS Power and Sample for Windows, version 3.1.6, with an independent t-test.

### Eligibility criteria

All participants were enrolled according to the settled eligibility criteria in general and for the teeth in specific, as presented in Table 1.

**Table 1:** Eligibility criteria [14, 15].

Eligibility criteria for participants	
Inclusion criteria	Exclusion criteria
• Cervical carious anterior lesions. • Age: (20–45Years). • Moderate caries risk patient according to the American Dental Association (ADA).	• Poor oral hygiene. • Unable to return for recall appointments • Presence of para-functional habits. • Systemic diseases interfere with participation in the study. • TMJ problems • Lack of compliance. • Rampant caries. • Xerostomia.
**Eligibility Criteria for Teeth**	
• Cervical carious lesion. (ICDAS score 4, Nyvad score three cavitated active lesions). • Dentin sclerosis scale 1 & 2, one (no sclerosis) or two (less than 50%) **(Perdigao et al., 2020)**. • Vital teeth. • Normal occlusion. • Accessible isolation. • The gingival margin of the lesion should be within the enamel. • VAS from 4-6	• Pulpitis. • Presence of NCCLs • Endodontically treated teeth. • Abnormal occlusion. • Periodontal disease may affect the prognosis of the restoration or the tooth itself. • Root Caries. • Violation of biological width • Deep cavity according to the electrical impedance readings.

Table 1: Eligibility criteria [20, 21].

### Recruitment, randomization, and allocation concealment

A total of 32 patients were screened for eligibility, of which 26 met the study’s inclusion criteria. The remaining patients did not meet the study’s participation criteria and were therefore excluded from the study. Twenty-six participants underwent a comprehensive examination and diagnosis. A total of 44 cervical carious lesions were allocated, with a maximum of two lesions per participant. After identifying potentially eligible participants for this study, the principal researcher provided a detailed explanation of the study and assessed the patients’ interest in participation. The patient signed an informed consent form indicating approval for all procedures and acceptance of participation in this trial. Randomization was done using simple randomization by (D.E.) with computerized sequence generation using www.random.org, generating numbers from 1:44 organized into two columns, with the lesion serving as the randomization unit. The randomization list was kept secure to prevent tampering with it. (O.H.) generated the random allocation sequence and assigned the intervention/ comparator identification procedures to the respective teeth. Each participant selected an opaque sealed envelope that contained the random allocation. Each envelope was signed, and the corresponding number was documented in the patient chart to ensure proper assignment.

### Participants preparation

Following the approval of consent, the main researcher conducted a preparatory appointment, which included prophylaxis. A thorough examination of all risk factors associated with caries occurrence was conducted to formulate a strategy for participants to improve their oral hygiene and mitigate any conditions that could compromise the study’s outcome. The presence of tooth sensitivity was evaluated based on the established inclusion criteria (VAS 4-6).

### Field preparation

Field preparation involved administering local anesthesia (Art-pharmadent 1:100,000 Articaine/epinephrine) to participants prior to lesion removal and preparation. A 330 bur (SS White, USA), measuring 0.8 mm in diameter and 1.6 mm in length, was utilized in a high-speed handpiece with air/water coolant to prepare class V cavity preparations featuring incisal and gingival margins in enamel. A suitable spoon excavator was employed to eliminate any soft caries (Dentsply, Switzerland). A yellow-coded tapered finishing stone (MANI, Japan) beveled all margins with a short 45^o^ angulation, except at the cervical margin. The depth of the cavity was arbitrarily assessed pre-operatively and confirmed after cavity preparation using a calibrated electrical impedance, the Prepometer (Hagar & Werken, Duisburg, Germany). The electrode was centrally placed inside a wet cavity, and a clip was attached to the participant’s cheek. It displays four colors: green, yellow, and orange, indicating that the cavity was not deep, unlike the red [22]. Sectional isolation was performed using suitable clamps from the second right to left premolar to better visualize the lesion with heavy sheets (Powder-free Latex Silk Blue Heavy Dental Dam, Sanctuary Health Company, Malaysia). Inversion and floss ligation were a part of the rubber dam application; a subgingival clamp was applied when necessary [20].

### Application of nitric acid and mineral enriched system (intervention)

For the intervention group, a multifunction nitric acid etch (Clean and boost dentin and enamel cleanser, Vista apex) was applied in three steps: first, a flow-through brush tip delivered the cleanser to ensure complete saturation of the surface; second, the cleanser was agitated for 10 s; and third, the surface was rinsed thoroughly for another 10 s. A single coat of the mineral-enriched adhesive (RE-GEN Universal Adhesive, Vista Apex) was applied using a microbrush in a rubbing motion for 10 s. Then, it was air-thinned for an additional 10 s. The bioactive flowable composite (RE-GEN Bioactive Flowable Composite, Vista Apex) was used as a liner on the bonded surfaces within the system. Then, the adhesive and flowable were co-cured to increase their bioactivity using a calibrated light-curing system (Woodpecker Light Cure I LED, China) with an intensity of 1600 mW/cm^2^ for 20 s, according to the manufacturer’s instructions. The irradiance was verified before each use with a radiometer to ensure consistent curing performance.

### Application of phosphoric acid with regular adhesive and flowable system (comparator)

Phosphoric acid **(Scotchbond Universal Etchant, 3 M)** was applied to enamel and dentin for 15 s. The surface was rinsed for 10 s and then gently air-dried to eliminate excess moisture without desiccating the dentin structure. Two coats of universal adhesive **(Scotchbond Universal Adhesive, 3 M) were applied actively with a microbrush for 20 s, with gentle air thinning** between the two layers and after application to allow solvent evaporation and optimize the adhesive-tooth interaction. Bonded surfaces were light-cured for 20 s using the same curing unit [21, 23]. A flowable composite **(Filtek Supreme, 3 M)** was applied as a liner over the dentin substrate and subsequently light-cured for 20 s [24].

### Final restoration for both groups

A nanohybrid resin composite (**Filtek Z350 XT, 3 M)** of appropriate shade was utilized to fill the cavities in one or two increments, depending on the size of each cavity. The shade was cross-matched using a customized shade guide made from the same material, ensuring consistency and accuracy. The buildup and sculpting were performed using the freehand technique by the tooth contour. Each restoration was finished using a red-coded tapered finishing stone (MANI, Japan) and a yellow one (MANI, Japan) in a high-speed handpiece under a water spray. The sequence of aluminum oxide finishing discs (TOR VM, Alex Dent) was used in descending order in a low-speed handpiece, as follows: coarse (70-90 µm), medium (40 µm), fine (24 µm), and superfine (8 µm) [20, 24]. Polishing was optimized using impregnated Eve tips. One operator (N.Y.) completed all restorative steps for standardization. All materials, along with their commercial names and specifications, are presented in Table 2.

**Table 2:** Materials’ names, specifications, composition, manufacturers & websites, and batch numbers.

Materials’ names	Specifications	Compositions	Manufacturers & Websites	Batch numbers
**Clean and boost dentin and enamel cleanser**	Nitric acid	• 2-hydroxyethyl methacrylate (1–10%), • Propan-2-ol (1–5%) and • Nitric acid (0.5–3%). pH ≈ 0.9	**Vista Apex, USA** https://www.vistaapex.com/	071522
**RE-GEN Universal adhesive**	Mineral-enriched adhesive	• Bioglass 45S5 (45 wt% SiO_2_, 24.5 wt% CaO, 24.5 wt% Na_2_O, 6 wt% P_2_O_5_), Fluoride • 2-Propenoic acid, 2-methyl-, (1-methylethylidene) bis [4,1 4,1-phenyleneoxy (2-hydroxy-3,1-propanediyl)] ester (15–40%), • Ethyl alcohol (15–40%), • Acetone (1–5%), • Bis [2- [(2-methyl oxoallyl) oxy] ethyl] dihydrogen benzene1,2,4, 5-tetracarboxylate (10–30%), • 2-hydroxyethyl methacrylate (10–30%), • 10-Methacryloyloxydecyl dihydrogen phosphate (10-MDP) from (7 to 13%), and • Benzoic acid, 4-(dimethylamino)-, ethyl ester Benzoic acid, 4-(dimethylamino)-, ethyl ester (0.5–1.5%). pH ≈ 3	**Vista Apex, USA** https://www.vistaapex.com/	20223343
**RE-GEN Bioactive Flowable Composite**	Bioactive flowable composite	• • Barium glass (40–80%), • 2-Propenoic acid, 2-methyl-, (1-methylethylidene) bis [4,1- phenyleneoxy(2-hydroxy-3,1-propanediyl)] ester (10-40%), • Triethylene glycol dimethacrylate (10–30%), • Submicron Silica (1–15%) and • Benzoic acid, 4-(dimethylamino)- with ethyl ester (0-2%).	**Vista Apex, USA** https://www.vistaapex.com/	20223055
**Scotchbond Universal Etchant**	Phosphoric acid etchant	• 34% phosphoric acid by weight pH ≈ 0.1. • Fumed silica and a water-soluble polymer to control the viscosity	**3 M ESPE, USA** https://www.3mlietuva.lt/3M/lt_LT/p/d/b5005223013/	19312001
**Scotchbond Universal adhesive**	Universal adhesive	• MDP Phosphate Monomer, • imethacrylate resins, • HEMA (Hydroxyethyl Methacrylate), • Vitrebond™ Copolymer, • Nanofillers, • Ethanol, Water, and Silane coupling agent. pH ≈ 2.7	**3 M ESPE, USA** https://www.3mlietuva.lt/3M/lt_LT/p/d/b5005223013/	30720 A
**Filtek Supreme Flowable Restorative**	Nanofilled flowable composite	• Procrylat, BisGMA (Bisphenol A glycerolate dimethacrylate), &TEGDMA (Triethylene glycol dimethacrylate). • A combination of a non-agglomerated/non-aggregated surface modified 20 nm silica filler, a non-agglomerated/non-aggregated surface modified 75 nm silica filler, a surface modified aggregated zirconia/silica cluster filler (comprised of 20 nm silica and 4 to 11 nm zirconia particles), and ytterbium trifluoride filler with a range of particle sizes from 0.1 to 5.0 μm. The aggregate has an average cluster particle size of 0.6–10 μm. The total inorganic filler loading is approximately 65% by weight (46% by volume).	**3 M ESPE, USA** https://www.3mlietuva.lt/3M/lt_LT/p/d/b5005223013/	10139439
**Filtek Z350 XT**	Nanohybrid packable composite	• Combination of aggregated Zirconia/Silica cluster with primary particle size (5–20 nm), and non-agglomerated silica filer (20 nm) & 78.5 Wt%, and • The organic matrix consists of Bis-GMA, UDMA (urethane dimethacrylate), TEGDMA, and Bis-EMA (ethoxylated bisphenol A dimethacrylate).	**3 M ESPE, USA** https://www.3mlietuva.lt/3M/lt_LT/p/d/b5005223013/	NG16860

Table 2: Materials’ names, specifications, compositions, manufacturers & websites, and batch numbers.

### Outcome assessment

- **Calibration of the assessors:**
  Calibration sessions for the assessors (OH and DE) were conducted one month prior to the assessment appointment and continued until a high degree of agreement was reached. Such calibration was calculated on 10 patients not included in the trial. In cases of disagreement, an extended, in-depth discussion was held until a consensus was reached. Intra- and inter-assessor agreement were tested using Cohen’s Kappa test, and it reached 100% after the calibration sessions. The assessors did not participate in any procedural steps to mitigate any risk of bias.
- **Primary outcome assessment using modified USPHS criteria and VAS:**
  The primary outcome assessed was postoperative sensitivity. Sensitivity was evaluated at 24 h and subsequently at 6, 12, and 18 months using two methods: the USPHS criteria and the VAS to capture patients’ subjective perception of sensitivity. According to the USPHS criteria, sensitivity was categorized as either Alpha or Charlie, depending on its absence or presence in response to thermal and air-drying stimuli (Table 3). VAS was used to provide a quantitative measure of sensitivity, offering additional insight into patient-reported outcomes. It is a horizontal line of 10 digits from 0 to 10 cm. Digit 0 indicates no pain, while digit 10 indicates severe pain. Each participant was subjected to air-drying and thermal stimuli. An air syringe from a conventional dental unit was used for evaporative sensitivity, positioned approximately 5 mm from the tooth surface. The air temperature was 25˚C ± 3˚C at a pressure of 0.5 N/mm^2^ [25]. The duration of the air blast ranged from 1 to 5 s, as reported by the participants. The stimulus was stopped when the participants reported pain, and pain intensity was recorded. The thermal stimuli were evaluated using an anesthetic carpule stored in a refrigerator at 4 °C for at least 24 h, until it had become ice; it was then removed immediately before testing. For thermal sensitivity assessment, the carpule was placed centrally on the tooth for 1–5 s, following the same protocol for evaporative testing [26]. Participants indicated their sensitivity level on a 10-cm visual analog scale (VAS), and the highest reported response (whether from evaporative or thermal stimuli) was recorded as the overall sensitivity score. Postoperative sensitivity was classified as follows: no pain (VAS = 0), mild pain (VAS 1–3), moderate pain (VAS 4–6), and severe pain (VAS 7–10) [27]. Mild and moderate sensitivity did not necessitate restoration replacement, as these were managed with analgesic prescriptions [28]. Patients are advised not to take any analgesic medications within 12 h prior to the assessment visit. This is essential to ensure an accurate evaluation of postoperative sensitivity and pain levels.
- **Secondary outcomes assessment using modified USPHS criteria:**

**Table 3:** Clinical assessment of the restorations using modified USPHS criteria.

Outcome		Score	Characteristics	Methods of diagnosis
**Primary**	**Postoperative sensitivity**	Alpha	Absence of postoperative sensitivity	Thermal using an iced anesthetic carpule and Evaporative using air steam directed toward the tooth restoration interface.
		Charlie	Presence of postoperative sensitivity	
**Secondary**	**Marginal discoloration**	Alpha	No discoloration	By visual inception
		Bravo	Shallow discoloration, which can be polished away	
		Charlie	Deep discoloration	
	**Marginal adaptation**	Alpha	No visible crevice or so small that the probe catches it and does not fall in.	By using a dental explorer
		Bravo	The explorer tip fell into a crevice, but the dentin was not exposed.	
		Charlie	The explorer penetrates a crevice that is of a depth that exposes dentin or base	
	**Secondary caries**	Alpha	Absence of caries	By visual inception
		Charlie	Presence of caries	
	**Retention**	Alpha	No loss of restorative material	By visual inception
		Charlie	Missing restoration	

The tested criteria were marginal discoloration, marginal adaptation, secondary caries, and retention (Table 3). They were assessed following the same time frame as the primary outcome. Marginal discoloration was evaluated by visually inspecting the interface between the restoration and the adjacent tooth structure for any staining. Scores were assigned as follows: Alpha (no discoloration), Bravo (slight discoloration not requiring replacement), or Charlie (discoloration requiring replacement of the restoration). Marginal adaptation was assessed by gently probing the restoration margins with an explorer to detect any gaps or irregularities. Criteria were defined as: Alpha (perfect adaptation with no detectable crevice), Bravo (detectable crevice that does not expose dentin), or Charlie (crevice exposing dentin or base, indicating restoration failure). Secondary caries was determined by the presence or absence of recurrent carious lesions adjacent to the restoration. Scores were Alpha (no evidence of caries) or Charlie (visible evidence of secondary caries necessitating restoration replacement). Retention was recorded as either Alpha (restoration fully retained) or Charlie (restoration partially or wholly lost). No intermediate (Bravo) score is typically used for this criterion in the USPHS system.

### Statistical analysis

Statistical analysis was conducted using Medcalc software, version 22 for Windows (Medcalc Software Ltd, Ostend, Belgium). Data were analyzed using an intention-to-treat analysis, following the last observation carried forward method. Categorical data of the modified USPHS criteria were described in terms of frequency and percentage. Inter-group comparisons between interventions were performed using the Chi-Squared test, and intragroup comparisons within each intervention were performed using Cochran’s Q test, followed by multiple pairwise comparisons. The Shapiro-Wilk test was employed to assess the normality of continuous data. Continuous data exhibited a non-parametric distribution, characterized by its minimum, maximum, median, and range. Intergroup comparison of VAS values was performed using the Mann-Whitney test. In contrast, intragroup comparisons within each treatment group were conducted using the Friedman test, followed by multiple comparisons with a statistical significance level (p ≤ 0.05). Spearman’s correlation was used to correlate between VAS and modified USPHS in the assessment of postoperative sensitivity. Relative risk was used to assess the clinical significance of the findings. The survival rate was analyzed using the Kaplan-Meier method and the Log-rank test. The confidence limit was set at 95% with 80% power, and all tests were two-tailed with a statistical significance level set at *p* ≤ 0.05.

## Results

### Demographic data

**Fig. 2:**
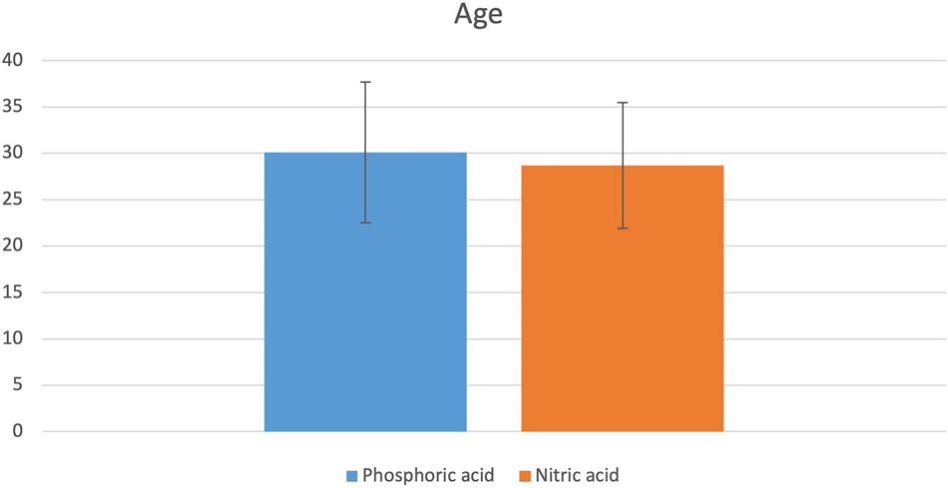
Demographic data. Bar chart showing age among both groups.

**Fig. 3:**
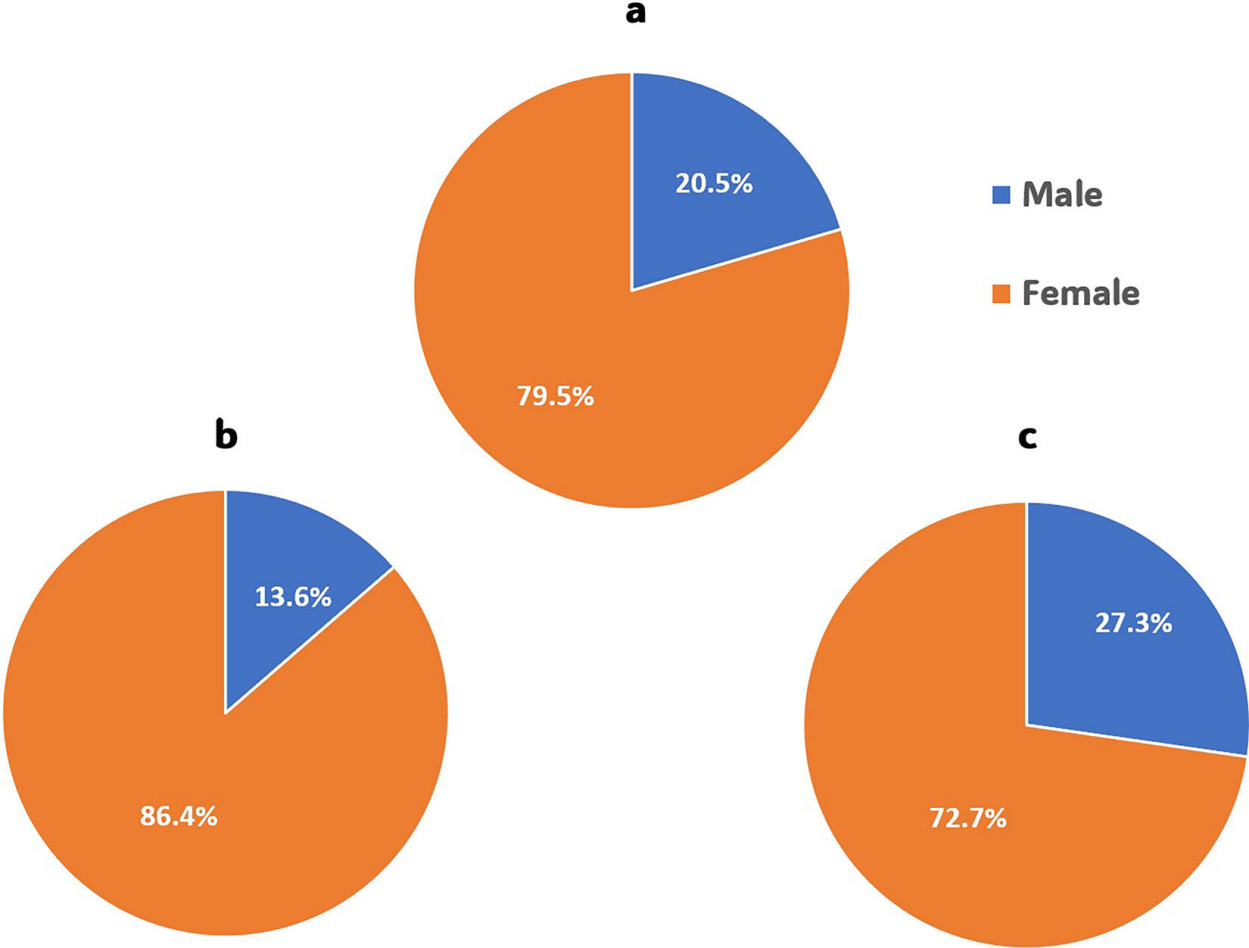
Demographic data showing gender distribution in precentage among groups. Pie charts showing the distribution; (**a**) in the whole study; (**b**) within the comparator; (**c**) within the intervention.

**Fig. 4:**
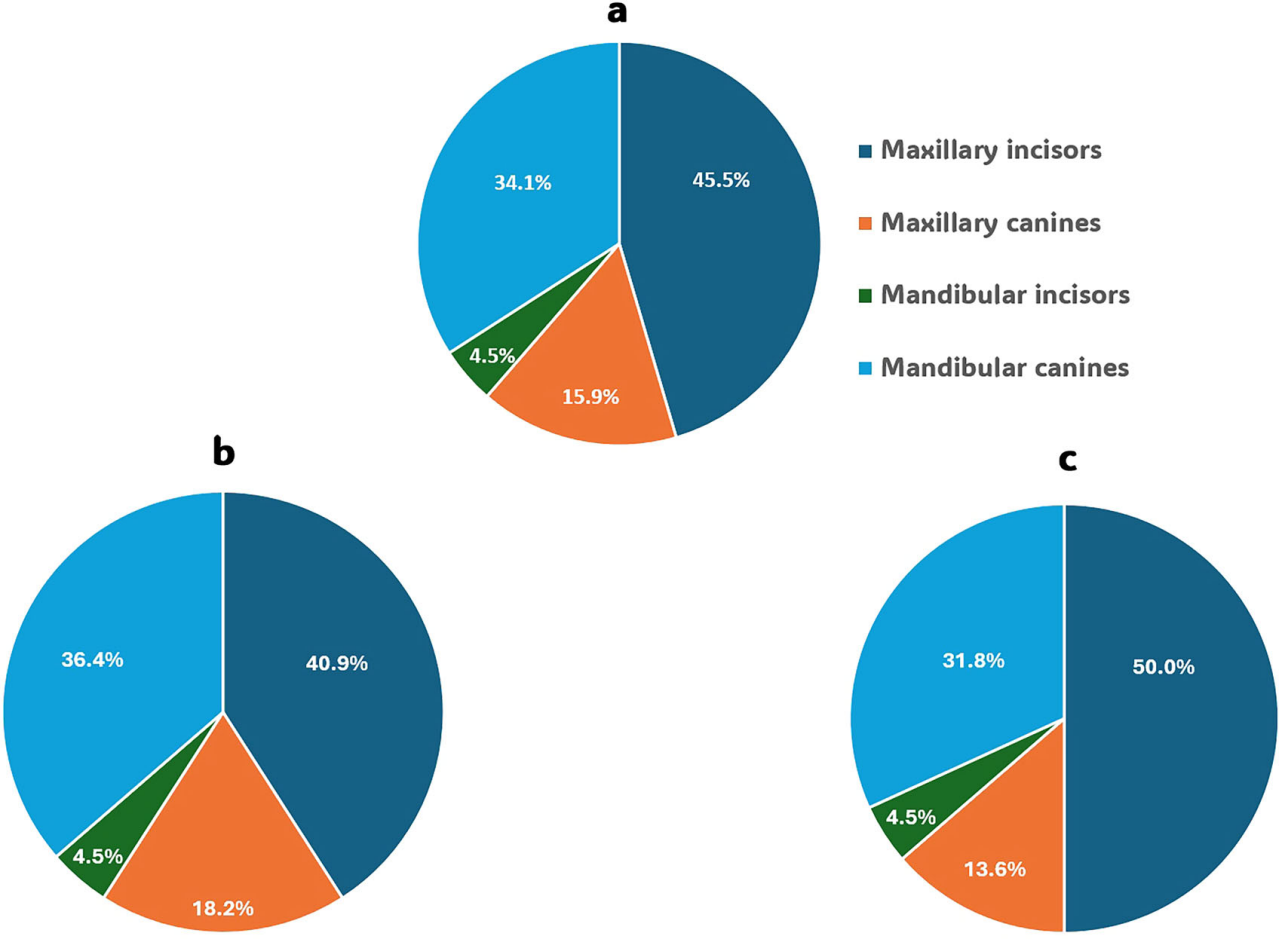
Demographic data showing teeth distribution in percentage among groups. Pie charts showing the distribution; (**a**) in the whole study; (**b**) within the comparator; (**c**) within the intervention.

This study was conducted on 26 participants with 44 cervical carious lesions, which were randomly allocated to the intervention and comparator arms (*n* = 22). After 18 months, 41 restorations were assessed with a 93.3% retention rate. Mean age of the participants in the current trial was 29.41 ± 7.2 years; mean age within nitric acid group was 28.7 ± 6.8 years, while within the phosphoric acid group mean age was 30.1 ± 7.6 years, there was no significant difference between both groups regarding age (*p* = 0.510) as shown in Fig. 2. Gender distribution indicated no significant difference between both groups (*p* = 0.2677) (Fig. 3). Regarding, teeth distribution, there was no significant difference between both groups (*p* = 0.9383) (Fig. 4).

### Modified USPHS criteria

#### Postoperative sensitivity

Table 4 An intergroup comparison between the two materials revealed no significant difference across various follow-up periods: 24 h, 6 months, 12 months, and 18 months (*p* > 0.05). Intragroup comparisons within nitric and phosphoric acid have shown no significant change through time (*p* = 0.801).

**Table 4:** Frequency and percentage of inter- and intragroup comparisons between materials within follow-up intervals regarding different tested criteria.

	Follow-up	Phosphoric acid		Nitric acid		*P* value		
		A	C	A	C			
Postoperative sensitivity	24 h	17 (77.3%)	5 (22.7%)	21 (95.5%)	1 (4.5%)	*P* = 0.0824		
	6 months	18 (81.8%)	4 (18.2%)	21 (95.5%)	1 (4.5%)	*P* = 0.1589		
	12 months	18 (81.8%)	4 (18.2%)	21 (95.5%)	1 (4.5%)	*P* = 0.1589		
	18 months	18 (81.8%)	4 (18.2%)	20 (90.9%)	2 (9.1%)	*P* = 0.3851		
	*P* value	*P* = 0.801		*P* = 0.801				
**Retention**	24 h	22 (100%)	0 (0%)	22 (100%)	0 (0%)	*P* = 1.0000		
	6 months	22 (100%)	0 (0%)	22 (100%)	0 (0%)	*P* = 1.0000		
	12 months	22 (100%)	0 (0%)	22 (100%)	0 (0%)	*P* = 1.0000		
	18 months	22 (100%)	0 (0%)	22 (100%)	0 (0%)	*P* = 1.0000		
	*P* value	*P* = 1.0000		*P* = 1.0000				
**Secondary caries**	24 h	22 (100%)	0 (0%)	22 (100%)	0 (0%)	*P* = 1.0000		
	6 months	22 (100%)	0 (0%)	22 (100%)	0 (0%)	*P* = 1.0000		
	12 months	22 (100%)	0 (0%)	22 (100%)	0 (0%)	*P* = 1.0000		
	18 months	22 (100%)	0 (0%)	22 (100%)	0 (0%)	*P* = 1.0000		
	*P* value	*P* = 1.0000		*P* = 1.0000				
**Marginal discoloration**	**Follow-up**	**A**	**B**	**C**	**A**	**B**	**C**	***P* value**
	24 h	22 (100%)	0 (0%)	0 (0%)	22 (100%)	0 (0%)	0 (0%)	*P* = 1.0000
	6 months	20 (90.9%)	2 (9.1%)	0 (0%)	20 (90.9%)	2 (9.1%)	0 (0%)	*P* = 1.0000
	12 months	17 (77.3%)	5 (22.7%)	0 (0%)	20 (90.9%)	2 (9.1%)	0 (0%)	*P* = 0.2216
	18 months	17 (77.3%)	5 (22.7%)	0 (0%)	18 (81.8%)	4 (18.2%)	0 (0%)	*P* = 0.7118
	*P* value	*P* = 0.007*			*P* = 0.112			
**Marginal adaptation**	24 h	22 (100%)	0 (0%)	0 (0%)	22 (100%)	0 (0%)	0 (0%)	*P* = 1.0000
	6 months	22 (100%)	0 (0%)	0 (0%)	22 (100%)	0 (0%)	0 (0%)	*P* = 1.0000
	12 months	22 (100%)	0 (0%)	0 (0%)	22 (100%)	0 (0%)	0 (0%)	*P* = 1.0000
	18 months	22 (100%)	0 (0%)	0 (0%)	21(95.5%)	1 (4.5%)	0 (0%)	*P* = 0.3173
	*P* value	*P* = 1.0000			*P* = 0.392			

#### Retention

An intergroup comparison between the two materials revealed no significant difference across the follow-up periods of 24 h, 6, 12, and 18 months (*p* > 0.05). Intragroup comparison within nitric acid and phosphoric acid has shown no significant change through time (*p* = 1.0000). There was no risk of retention loss (score C) with nitric acid when compared to phosphoric acid after 18 months (RR = 1.0000 (95% 0.02071–48.2867; *p* = 1.0000)).

#### Secondary caries

An intergroup comparison between the two materials revealed no significant difference across the follow-up periods of 24 h, 6, 12, and 18 months (*p* > 0.05). Intragroup comparison within nitric acid and phosphoric acid has shown no significant change through time (*p* = 1.0000). There was no risk of secondary caries (score C) with nitric acid when compared to phosphoric acid after 18 months (RR = 1.0000 (95% 0.02071 to 48.2867; *p* = 1.0000)).

#### Marginal discoloration

An intergroup comparison between the two materials revealed no significant difference across the follow-up periods of 24 h, 6 months, 12 months, and 18 months (*p* > 0.05). An intragroup comparison within nitric acid has shown no significant change over time (*p* = 0.112). An intragroup comparison within phosphoric acid has shown a statistically significant change over time (*p* = 0.007). There was 25% more risk of marginal discoloration (score B) within phosphoric acid when compared to nitric acid after 18 months (RR = 1.25 (95% 0.3862 to 4.0457; *p* = 0.7096)). However, this result was not statistically significant as the *p* value exceeds 0.05% and the confidence interval crosses 1.0.

#### Marginal adaptation

An intergroup comparison between the two materials revealed no significant difference across the follow-up periods of 24 h, 6 months, 12 months, and 18 months (*p* > 0.05). Intragroup comparison within nitric acid has shown no statistically significant difference between different follow-up periods (*p* = 0.392). Intragroup comparison within phosphoric acid has shown no significant difference between different follow-up periods (*p* = 1.0000). There was 3 times more risk for marginal adaptation (score B) within nitric acid when compared to phosphoric acid after 18 months (RR = 3.0000 (95% 0.1288–69.8718; *p* = 0.4940)). However, this result was not statistically significant as the *p* value exceeds 0.05% and the confidence interval crosses 1.0.

#### Quantitative results for postoperative sensitivity using VAS

**Fig. 5:**
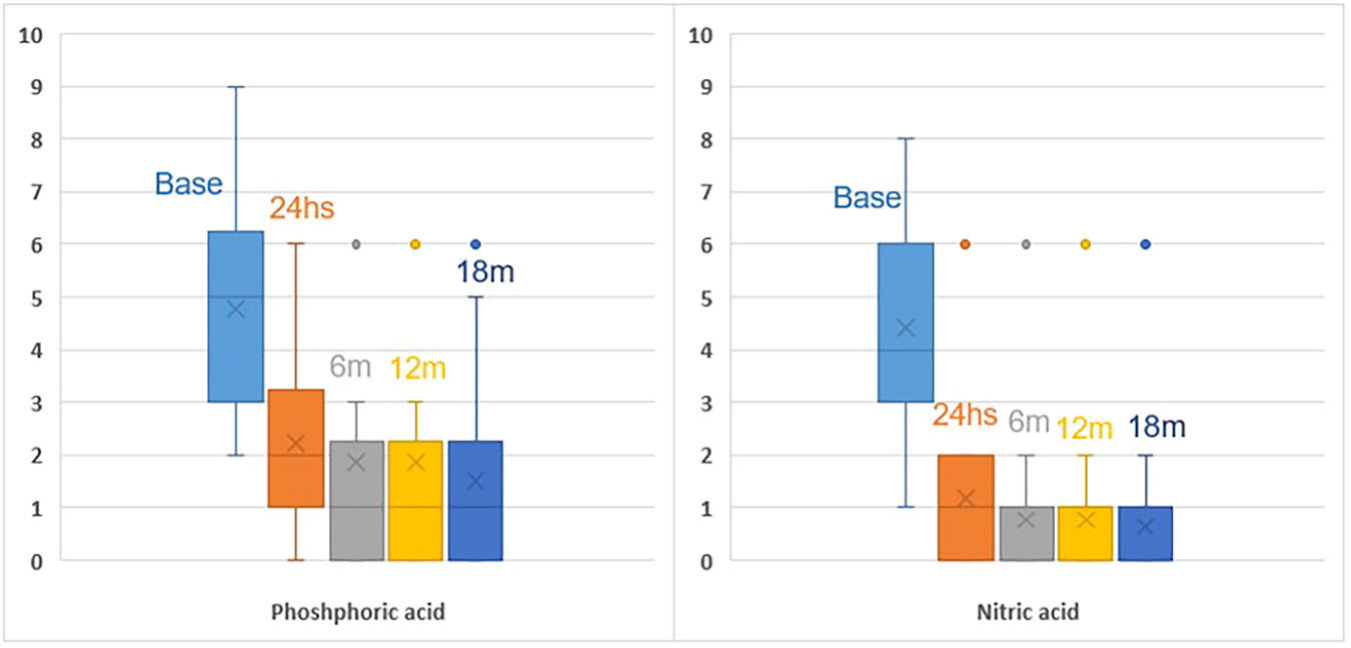
Quantatative assessment of postoperative sensitivity. Box plot showing mean VAS within each material at different follow-up peroids.

An intergroup comparison between the two materials revealed no statistically significant difference before the intervention (*p* = 0.6438). However, after 24 h, 6, 12, and 18 months, a significant difference was observed (*p* < 0.05). Intragroup comparison within nitric acid has shown a significant change in postoperative sensitivity through time (*p* < 0.00001). Intragroup comparison within phosphoric acid has shown significant change in sensitivity through time (*p* < 0.00001) (Fig. 5). The median change after 18 months in VAS in nitric acid group was –3.5 (95% CI –4.5 to –3.0), while the median difference within phosphoric acid group was -3.5 (95% CI –4 to –2.5).

#### Categorical results for postoperative sensitivity

**Fig. 6:**
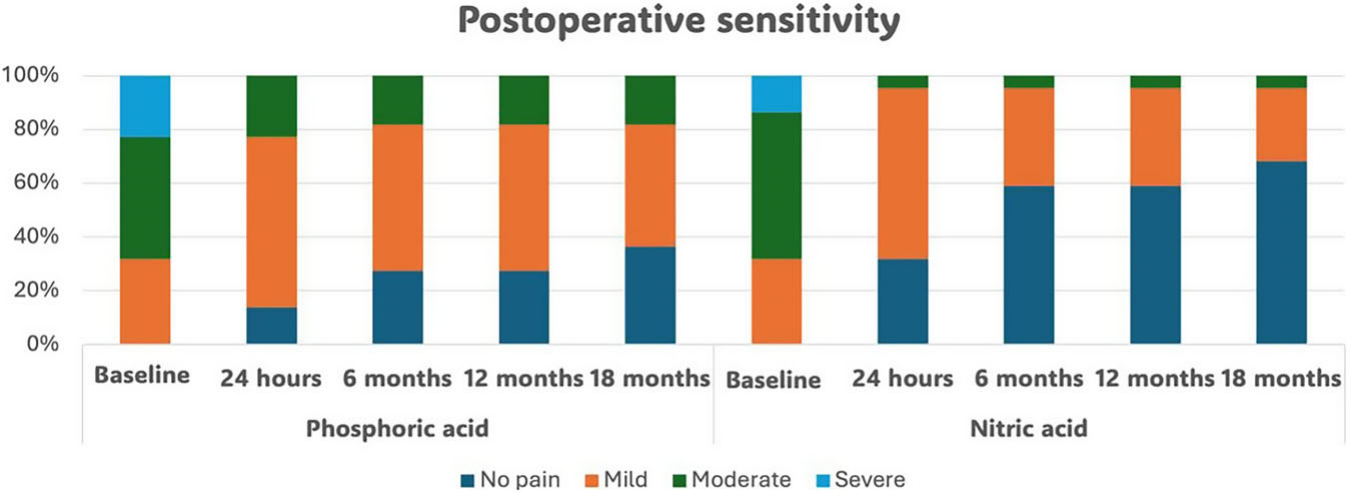
Categorical assessement of postoperative sensivity. 100% stacked column chart showing percentage of different scores within each material at different follow-up peroids.

**Fig. 7:**
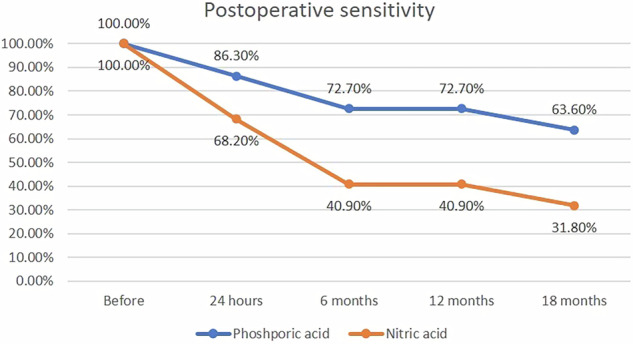
Postoperative sensitivity through time. Line chart showing change in sensitivity percentage among participants within both groups.

An intergroup comparison between the two materials revealed no significant difference before intervention (*p* = 0.7111). However, at 24 h, 6, 12, and 18 months of follow-up, a significant difference was observed (*p* < 0.05). Intragroup comparison within nitric and phosphoric acid has shown significant improvement through time (*p* < 0.001 and 0.008, respectively) (Table 5 and Fig. 6, 7). The success rate was calculated by considering no pain as success and considering mild, moderate, and severe pain as failure. Therefore, the success rate of phosphoric acid was 36.4% and the success rate of nitric acid was 68.2% in the management of postoperative sensitivity. There was 50% less risk of sensitivity with nitric acid when compared to phosphoric acid after 18 months (RR = 0.5(95%CI 0.2512–0.9953; *p* = 0.0485)).

**Table 5:** Frequency and percentage for postoperative sensitivity scores for the inter and intragroup comparison within each follow-up intervals.

Follow-up	Phosphoric acid				Nitric acid				*P* value
	No pain	Mild	Moderate	Severe	No pain	Mild	Moderate	Severe	
Before	0 (0%)	7 (31.8%)	10 (45.5%)	5 (22.7%)	0 (0%)	7 (31.8%)	12 (54.5%)	3 (13.6%)	*P* = 0.7111
24 h	3 (13.6%)	14 (63.6%)	5 (22.7%)	0 (0%)	7 (31.8%)	14 (63.6%)	1 (4.5%)	0 (0%)	*P* = 0.0431*
6 months	6 (27.3%)	12 (54.5%)	4 (18.2%)	0 (0%)	13 (59.1%)	8 (36.4%)	1 (4.5%)	0 (0%)	*P* = 0.0237*
12 months	6 (27.3%)	12 (54.5%)	4 (18.2%)	0 (0%)	13 (59.1%)	8 (36.4%)	1 (4.5%)	0 (0%)	*P* = 0.0237*
18 months	8 (36.4%)	10 (45.5%)	4 (18.2%)	0 (0%)	15 (68.2%)	6 (27.3%)	1 (4.5%)	0 (0%)	*P* = 0.0277*
*P* value	*P* = 0.008*				*P* < 0.001*				

#### Success rate of modified USPHS criteria

**Fig. 8:**
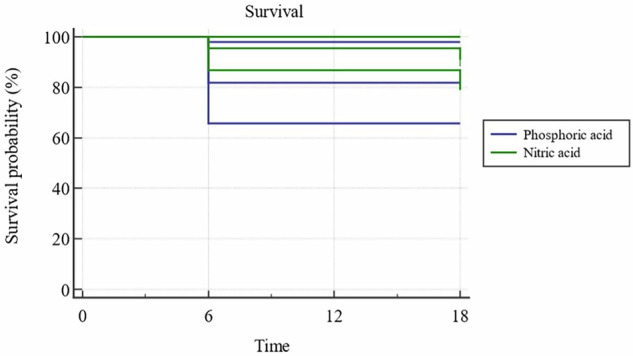
Survival rate. Analysis of phosphoric and nitric acids after 18 months.

After 18 months, cervical restorations in the nitric acid group showed a 90.9% success rate. In contrast, the phosphoric acid group achieved an 81.8% success rate, primarily due to scoring Charlie for postoperative sensitivity. There was 50% less risk for failure of nitric acid when compared to phosphoric acid using USPHS criteria (RR = 0.5 (95% (0.1018–2.4549); *p* = 0.3932)), yet this difference was not statistically significant (*p* = 0.3851). Kaplan-Meier analysis was used to obtain survival curves, and a comparison of survival curves was performed using the Log-rank test; there was no significant difference between the two materials (*p* = 0.3771) (Fig. 8).

## Correlation

**Fig. 9:**
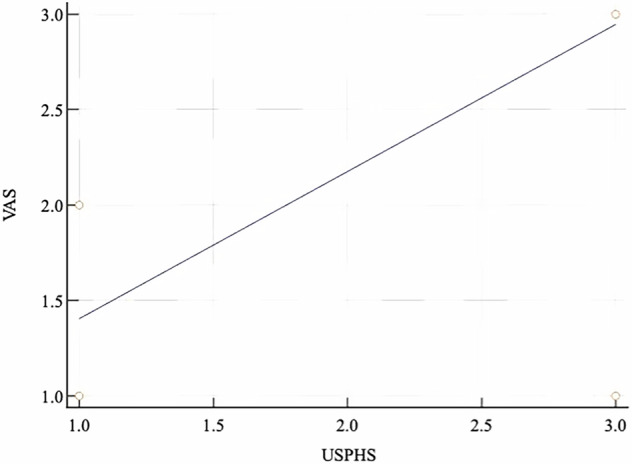
Correlation between quantitative and qualitative assessements of postoperative sensitivity. Scatter diagram showing moderate to strong correlation.

There was a moderate to strong positive correlation between assessments of postoperative sensitivity using quantitative (VAS) and qualitative (USPHS) methods after 24 h, 6, 12, and 18 months; this correlation was statistically significant (*p* < 0.05), as shown in Fig. 9.

## Discussion

The adhesive system plays a pivotal role in achieving durable cervical resin composite restorations by establishing intimate contact between the tooth structure and the resin composite. Various adhesives have been developed, culminating in the multimode adhesive, applicable in both etch-and-rinse (ER) and self-etch modes. Nevertheless, the optimal bonding strategy that ensures the best clinical outcomes remains undetermined [29]. Each mode possesses distinct advantages and disadvantages. The etch-and-rinse mode represents the oldest and most widely employed adhesive technique in dentistry, utilizing phosphoric acid as an etching agent at a concentration of 35–37%. The application occurs on both enamel (15–30 s) and dentin (10–15 s), leading to demineralization of the enamel surface through the extraction of mineral content, particularly calcium and phosphate, resulting in a microscopically rough surface. The removal of the smear layer and smear plug from the dentin surface generates micropores that facilitate the penetration of the bonding agent, thereby creating a connection between the restoration and the tooth structure. This mode establishes a robust micromechanical bond with enamel and dentin. However, it may elevate the risk of postoperative sensitivity, particularly when the dentin becomes etched and there is an increased possibility of enzymatic activity, which can jeopardize the durability of the restoration [30, 31].

Postoperative sensitivity, particularly following application of resin composite restorations, is a common complication that can significantly disrupt patients’ daily activities, reduce overall treatment satisfaction, and potentially compromise pulp vitality over time. Additionally, prolonged or severe sensitivity may contribute to decreased patient satisfaction and, in some cases, could potentially jeopardize pulp vitality over time. This pitfall could be attributed not only to the acid etch application but also to numerous other reasons, including the dentist’s skills, methods of cavity preparation, adhesive system, restorative materials used, and the age of the participants [8]. Numerous systematic reviews and randomized controlled trials consistently demonstrate that universal adhesives applied in etch-and-rinse mode provide superior clinical outcomes compared to the self-etch (SE) approach [12, 24, 29]. A 2020 meta-analysis of 13 clinical studies (2516 restorations) reported significantly higher retention (OR = 0.35) and better marginal adaptation (OR = 0.49). It reduced marginal staining (OR = 0.49) for ER protocols, with no significant differences in postoperative sensitivity or secondary caries between modes [29]. A 2023 network meta-analysis, including 16 RCTs ( > 2,800 restorations, ≥ 12-month follow-up), confirmed that ER mode consistently outperforms SE regarding marginal discoloration, adaptation failure, and fractures, while outcomes for ER and selective enamel-etch modes were comparable. Biological outcomes remained similar across all approaches [32]. Complementing these reviews, a 5-year RCT on Scotchbond Universal in non-carious cervical lesions found retention rates of 93% with ER versus 81% with SE (*p *= 0.01), and significantly better marginal adaptation and discoloration with ER [33]. Another meta-analysis, conducted by Reis et al. in 2015 [34], concluded that the type of adhesive strategy for resin composite posterior restorations did not influence the risk of postoperative sensitivity at different evaluation times.

While clinical evidence favors the etch-and-rinse application mode of universal adhesives for superior retention, marginal integrity, and esthetic performance, concerns persist regarding the long-term durability of the bond with this approach, particularly at the dentin interface. Several studies have highlighted that the complete removal of the smear layer and demineralization of the dentin surface during ER procedures exposes a thicker collagen matrix. This collagen network, if not adequately infiltrated and sealed by the adhesive monomers, becomes highly vulnerable to hydrolytic and enzymatic degradation over time, primarily due to the activation of matrix metalloproteinases (MMPs) and other endogenous enzymes [35]. This bond degradation concern contrasts with the excellent short and medium-term clinical outcomes observed with etch-and-rinse mode universal adhesives. The paradox is partly resolved by understanding that many contemporary universal adhesives are formulated with functional monomers such as 10-MDP, which can form stable chemical bonds with hydroxyapatite and may help preserve the hybrid layer even in a moist dentin environment. Moreover, many adhesives incorporate MMPS inhibitors (e.g., bioactive glass), which potentially mitigate enzymatic degradation and enhance long-term performance [36]. Therefore, while etch-and-rinse adhesives are theoretically more prone to bond degradation, clinical studies and meta-analysis show that when modern universal adhesives are applied correctly with proper solvent evaporation and light curing, they can still outperform self-etch modes in terms of retention and marginal adaptation, even after 5 years [29].

In light of the limitations associated with conventional adhesive systems, a novel approach has been proposed that involves the development of a bioactive adhesive system in conjunction with an alternative etching material. These challenges and emerging innovations formed the basis for the present study. This innovative strategy aims to enhance the bonding quality of both enamel and dentin by promoting chemical interaction, improving micromechanical retention, and supporting remineralization at the adhesive interface [37]. The use of bioactive components introduces therapeutic functionality, such as ion release and pH modulation, thereby potentially increasing the durability and biocompatibility of the adhesive-dentin complex. Upon contact with body fluids or simulated body fluid, bioactive glass undergoes immediate ionic dissolution and structural degradation. This process involves the exchange of hydrogen ions (H^+^) from the surrounding solution with sodium (Na^+^) and calcium (Ca^2+^) ions from the glass matrix. As a result, silanol groups (Si–OH) are formed through the hydrolysis of silica components. The release of hydroxide ions (OH^−^) increases the local pH, which accelerates degradation of the silica network and leads to the formation of orthosilicic acid (Si (OH)_4_), appearing as a negatively charged gel layer on the surface. This gel layer serves as a nucleation matrix for hydroxyapatite, enabling the precipitation of amorphous calcium phosphate and promoting remineralization [38]. These surface modifications are designed to improve the interaction between the adhesive and collagen fibers, contributing to the formation of a stable hybrid layer. The presence of the 10-MDP monomer in universal adhesives facilitates this process by chemically bonding with calcium ions in hydroxyapatite [39].

Historically, nitric acid was employed for etching both enamel and dentin in the late 1940s, although it was initially considered too aggressive because it caused excessive demineralization, rendering bonding unpredictable. Nevertheless, nitric acid has recently been reintroduced as an alternative etchant to phosphoric acid, with a reduced concentration (up to 3%) and shorter application time (10–15 s) [40]. The mechanism of action of nitric acid as an etchant is distinct from that of phosphoric acid, as it involves chemical dissolution and surface modification. The application of nitric acid superficially demineralizes the dentin, exposing the collagen and preserving the calcium ions needed for chemical bond with the 10 MDP monomer [41]. Additionally, it induces surface modification through its interaction with calcium ions to form calcium nitrate and hydroxide, which contributes to sealing the dentinal tubules and reducing dentin hydrophilicity. The selective demineralization of the enamel surface using nitric acid facilitates the formation of a more porous surface, thereby enhancing the mechanical retention of the adhesive [42]. The use of nitric acid in a mild concentration and for a brief period is considered a less devastating approach to dealing with the dentin substrate [43]. Such modes of action of nitric acid with the tooth substrates increased the long-term stability of the resin composite restoration [41].

Using bioactive glass in dental adhesives aims to enhance the bond strength and longevity of dental composite restorations. Its zinc content can protect the collagen matrix from degradation by matrix metalloproteinases (MMPs), which are activated by the acid etching step [44]. Moreover, bioactive glass can reduce micro permeability by remineralizing demineralized areas and enhancing the modulus of elasticity and hardness of the adhesive interface. Additionally, it decreases micro permeability by remineralizing areas deficient in minerals and enhancing both the modulus of elasticity and hardness. This process reduces dentin hypersensitivity by occluding the dentinal tubules through the binding of collagen fibers and the precipitation of hydroxyapatite. A review of the existing literature reveals a significant gap in clinical trials examining the effects of bioactive adhesives on postoperative sensitivity. A clinical trial conducted in 2002 was the sole relevant study identified, which investigated the bioactive system comprising S-PRG (Surface Pre-reacted Glass Ionomer) and its impact on postoperative sensitivity. The study findings demonstrate that the bioactive system effectively reduces postoperative sensitivity, which is linked to the release of six key ions: sodium, boron, aluminum, silicon, strontium, and fluoride [45].

The current study utilized nitric acid with a mineral-enriched adhesive and a flowable composite liner as the intervention arm. In contrast, the comparator arm used phosphoric acid and universal adhesive in etch-and-rinse mode, with a nanofilled flowable composite frequently used as a restorative system. The bioactive liner was used as recommended by Vista Apex to optimize the performance of the adhesive in the intervention arm. On the other hand, a regular flowable composite was used with the non-bioactive adhesive in the comparator arm to standardize the restorative protocol. The manufacturer of the cleanser and mineral-enriched system asserts that this protocol minimizes postoperative sensitivity using nitric acid etch, enhances bonding by attracting ions, and protects and heals the substrate. Bioglass facilitates the attraction and exchange of bioactive ions (Ca^2+^, PO_4_^3-^, F^-^) with the oral environment and tissue fluids, supported by continuous pH buffering. This mechanism helps prevent erosion and secondary decay, contributing to the closure of marginal gaps and maintaining marginal integrity. That is why postoperative sensitivity was taken as the primary outcome, and it was assessed both quantitatively using the VAS scale (either numerical or categorical) and qualitatively using the modified USPHS criteria. While marginal discoloration, marginal adaptation, secondary caries, and retention were evaluated using the modified USPHS criteria as secondary outcomes. Regarding the analysis of demographic data for the included participants, there was a homogeneous distribution among both groups in terms of age, gender, and teeth distribution (Figs. 2, 3, 4).

The results of the current study are inconclusive regarding the null hypothesis, which stated that there would be no difference between the two systems. A statistically significant difference was found between the intervention and comparator groups in the quantitative assessment of postoperative sensitivity. In contrast, no clinically significant differences were observed in the outcomes assessed by the modified USPHS criteria. They indicate that nitric acid and mineral-enriched systems demonstrated lower postoperative sensitivity than phosphoric acid, as measured by VAS. Additionally, the postoperative sensitivity decreased over time within each group. The results (Tables 4 and 5, and Fig. 5) may be attributed to the HEMA content in the cleanser, which, according to the manufacturer (Vista Apex, USA), is claimed to block dentin tubules and reduce postoperative sensitivity [46]. However, this interpretation remains speculative due to the lack of supporting clinical trials, and the explanation is primarily based on the manufacturer’s claims. Bio-regenerative mineral-enriched adhesives and flowable liners may reduce postoperative sensitivity by enhancing interfacial remineralization and optimizing bond quality [47]. This effect might also be supported by the dual action of acetone and ethanol solvents in the adhesive, which play a significant role in improving bond integrity. A combination of acetone and ethanol as solvents optimizes both monomer infiltration and solvent evaporation behavior during bonding procedures. Acetone, due to its high volatility and strong affinity for water, is particularly effective at displacing moisture from the demineralized dentin matrix, allowing better resin penetration into the exposed collagen network [48]. However, its rapid evaporation can make the adhesive more technique-sensitive, especially if solvent evaporation is incomplete. Ethanol, in contrast, is less volatile and evaporates more slowly, providing extended working time and enhanced stability of the adhesive solution [30]. When combined, these solvents complement each other: acetone improves water-chasing ability, while ethanol enhances monomer solubility and stabilizes the adhesive interface, especially in adhesives that include both hydrophilic (e.g., HEMA) and hydrophobic components [49]. Moreover, the dual-solvent strategy helps reduce the risk of phase separation within the adhesive, improving overall polymerization efficiency and bond strength, particularly when applied to moist dentin. Therefore, the use of both acetone and ethanol in dental adhesives offers a balanced formulation that enhances bonding efficacy while minimizing the challenges associated with single-solvent systems.

Postoperative sensitivity following resin composite restoration typically decreases over time through multiple interconnected biological mechanisms that facilitate healing and neural adaptation. The initial sensitivity primarily stems from the polymerization shrinkage of the composite material, which creates interfacial stresses and potential microleakage at the restoration margins, leading to fluid movement within exposed dentinal tubules and subsequent stimulation of intradental nerve endings, as per the hydrodynamic theory [50]. As healing progresses, several concurrent processes contribute to reduced sensitivity: dentinal tubule occlusion occurs through the natural precipitation of calcium and phosphate ions from dentinal fluids, the formation of smear plugs, and the deposition of mineral deposits, effectively reducing fluid flow and neural stimulation. Concurrently, the dental pulp initiates a protective response by forming secondary dentin at the restoration interface, providing enhanced insulation between the restoration and pulpal tissues [51]. The inflammatory response triggered by the etching and bonding procedures gradually subsides as residual acidic components are neutralized and unreacted monomers are cleared from the restoration interface, allowing normal pulpal circulation to resume [30]. Additionally, the restoration itself undergoes continued maturation as polymerization proceeds beyond the initial curing phase, improving the marginal seal and reducing microleakage. At the same time, the slight hygroscopic expansion of the composite material helps to close interfacial gaps [52]. Neural adaptation also plays a significant role, as the intradental nerve fibers gradually become less responsive to stimuli through desensitization mechanisms, ultimately resulting in the clinical resolution of postoperative sensitivity, which is typically observed within 2-4 weeks post-restoration [53].

RE-GEN is a Bisphenol A (BPA)- free, mineral-enriched dental adhesive designed to optimize both biological safety and clinical performance. Its BPA-free formulation eliminates concerns associated with estrogenic activity and cytotoxicity typically linked to bisphenol A-containing materials, making it more biocompatible for long-term use. Additionally, the absence of BPA enables a more stable and efficient integration of bioactive components, such as calcium and phosphate ions, which contribute to the adhesive’s remineralizing potential. The modified resin matrix used in RE-GEN offers favorable flow characteristics and enhanced polymerization, resulting in improved dentin infiltration and a more consistent hybrid layer. These features, combined with its mineral content, promote enhanced bond durability and the ability to support ongoing dentin repair. As such, RE-GEN’s BPA-free composition plays a central role in advancing both the safety and functionality of adhesive systems in restorative dentistry [54].

Cavity depth is a crucial factor to consider when evaluating postoperative sensitivity, as it ranges from shallow to medium and deep. It is essential to standardize this measurement in clinical trials. Shallow and medium cavities exhibit a favorable pulp reaction due to the ample dentin available to protect the pulp [55]. In the present study, deep cavities were excluded to avoid deviation in the obtained results. Furthermore, only one experienced operator performed all the standardized procedural steps for all participants, thereby minimizing the risk of bias.

Although there is a paucity of clinical trials evaluating the performance of nitric acid and mineral-enriched systems, previous studies by Francis et al. (2020) and Javed et al. (2024) reported similar postoperative sensitivity outcomes between etch-and-rinse and self-etch modes. This finding contrasts with our results [56, 57]. This discrepancy may be due to variations in the comparator groups; our study utilized a nitric acid-based bioactive system rather than a self-etch adhesive. In respect of marginal discoloration results (Table 4), phosphoric acid showed a higher bravo score after 12 and 18 months in comparison to nitric acid, and this could be explained by the antiplaque activity of the bioactive adhesive, which minimizes the accumulation of the chromogenic bacteria at the tooth restoration interface [58, 59]. Oz et al.^b^ [60] in 2019 presented distinct findings, as no cases of discoloration were observed in the phosphoric acid group after 24 months. They attributed this to the higher bonding ability of the adhesive used in the etch-and-rinse mode. The contradiction here could be due to the difference in the comparator group, as we use a bioactive adhesive system. Regarding the remaining assessed criteria, marginal adaptation, secondary caries, and retention, both systems yielded equivalent results, with a predominance of alpha scores (Table 4). Finally, an overall equal success rate is proven to document the clinical performance of the nitric acid cleanser and mineral-enriched system under the circumstances of the present study. Akimoto et al. [61] in 2011evaluated clinically a fluoride-releasing bioactive adhesive system incorporating S-PRG technology. The study demonstrated favorable outcomes, highlighting benefits such as the inhibition of secondary caries and the promotion of remineralization. However, the investigation did not include a direct comparison with a non-bioactive adhesive system, nor did it employ an adhesive formulation comparable to that used in the present study. Consequently, while the findings support the clinical effectiveness of the bioactive system, they do not provide comparative data that could confirm or contradict the current results. Instead, the study reinforces the general potential of bioactive adhesives in restorative dentistry.

One of the limitations of this study is the lack of a comprehensive assessment of the entire bioactive system in its full complexity. Future research should focus on a comprehensive evaluation of the complete bioactive system to elucidate the synergistic effects of all components and their overall performance. It is also important to note that this study focused on patients with moderate caries risk; high-risk patients may present unique challenges that could affect treatment outcomes, indicating that our findings may not apply to this population.

## Conclusions

According to the framework of this study, the following conclusions are drawn:

1. The use of nitric acid combined with a mineral-enriched system shows significant promise in mitigating postoperative sensitivity in patients undergoing resin composite restorations for cervical carious lesions.
2. The bioactive system, demonstrating clinical performance comparable to the non-bioactive counterpart, indicates favorable potential for its use in restorative dentistry.

### Recommendations

1. The combination of a final bioactive restorative material beside the mineral-enriched system used might be of clinical significance.
2. Additional clinical trials are needed to assess postoperative sensitivity in diverse clinical scenarios, including variations in lesion depth, sclerosis degree, lesion type, tooth location (with differing functional demands), and other patient-related factors.
3. Further laboratory-based studies are needed to clarify the underlying mechanisms of the RE-GEN system bioactivity.
4. Moreover, in vitro studies should also investigate the role of nitric acid-based Clean & Boost in modifying the tooth substrate to enhance bonding effectiveness.

## Data Availability

The datasets used and analyzed during the current study are available from the corresponding author on reasonable request.
